# Real-Time Ultrasound Diagnosis of Developmental Dysplasia of the Hip Using an Attention-Enhanced YOLOv11 Model

**DOI:** 10.7150/ijms.120138

**Published:** 2025-10-01

**Authors:** Wen-Shin Hsu, Guang-Tao Lin, Wei-Hsun Wang

**Affiliations:** 1 Department of Medical Information, Chung Shan Medical University, Taichung 402201, Taiwan.; 2 Informatics Office Technology, Chung Shan Medical University Hospital, Taichung 402201, Taiwan.; 3 Program for Precision Health and Intelligent Medicine, Graduate School of Advanced Technology, National Taiwan University, Taipei 106319, Taiwan.; 4 Department of Post-Baccalaureate Medicine, College of Medicine, National Chung Hsing University, Taichung 402202, Taiwan.; 5 Department of Golden-Ager Industry Management, Chaoyang University of Technology, Taichung 413310, Taiwan.; 6 Department of Orthopedic Surgery, Changhua Christian Hospital, Changhua 500209, Taiwan.; 7 Department of Medical Imaging and Radiology, Shu-Zen Junior College of Medicine and Management, Kaohsiung 821, Taiwan.

**Keywords:** Developmental Dysplasia of the Hip (DDH), Ultrasound Imaging, Deep Learning, YOLOv11, Medical Image Classification, Automated Diagnosis.

## Abstract

Developmental dysplasia of the hip (DDH) is a common pediatric orthopedic disorder that can lead to lifelong disability if undetected. Ultrasound is the primary diagnostic modality but is subject to operator dependence and inter-observer variability. To address this challenge, we propose an attention-enhanced YOLOv11 framework for automated DDH classification. A dataset of 6,075 hip ultrasound images was preprocessed with augmentation and dimensionality reduction via UMAP. The model integrates Cross-Stage Partial (CSP) modules and C2PSA spatial attention to improve feature extraction, and was trained using Focal Loss and IoU Loss. It achieved 95.05% accuracy with an inference speed of 11.5 ms per image, substantially outperforming MobileNetV3 and ShuffleNetV2. Grad-CAM visualizations confirmed that the model consistently attends to the acetabular roof and femoral head, landmarks central to Graf classification, thereby enhancing clinical interpretability. These findings demonstrate that the proposed framework combines technical robustness with clinical relevance. Future work will emphasize multi-center validation and multimodal integration to ensure generalizability and support widespread clinical adoption.

## 1. Introduction

Developmental Dysplasia of the Hip (DDH) is a prevalent pediatric orthopedic disorder that impairs hip joint development in infants 1. If undiagnosed or untreated, DDH may progress to hip joint instability, impaired mobility, and early-onset osteoarthritis, ultimately diminishing quality of life. Timely and accurate diagnosis is therefore essential to enable early intervention and prevent long-term complications. Ultrasound, particularly the Graf classification system, remains the standard diagnostic modality, as it assesses hip joint alignment through key anatomical angles 2. However, this approach requires substantial clinical expertise, rendering it highly susceptible to inter-observer variability 3. Moreover, variations in image quality and infant positioning further complicate interpretation, often leading to diagnostic inconsistencies 4. Given the high prevalence of DDH and the limitations of manual ultrasound interpretation, there is a pressing need for an automated, deep learning-based classification system to enhance diagnostic accuracy and efficiency.

Recent advances in deep learning have shown substantial promise in automating medical image analysis 5. Convolutional Neural Networks (CNNs), particularly architectures such as U-Net and Fully Convolutional Networks (FCNs), have achieved high accuracy in segmentation tasks across diverse medical imaging modalities 6. Nevertheless, most existing deep learning approaches for DDH diagnosis have focused on segmentation rather than direct classification, and their success often relies on large, expertly annotated datasets that are both costly and time-consuming to obtain 7. Furthermore, CNN-based models must contend with challenges such as dataset imbalance, variability in image quality, and limited availability of labeled samples 8. To address these challenges, this study introduces an automated DDH classification framework based on YOLOv11, a state-of-the-art real-time object detection model 9. Unlike conventional CNN-based segmentation networks, YOLOv11 provides an efficient and unified approach to simultaneously detecting and classifying DDH-related hip structures in ultrasound images, thereby improving diagnostic accuracy and consistency 10. In addition, the framework leverages data augmentation to mitigate class imbalance and employs UMAP for dataset visualization 11. By integrating advanced attention mechanisms and optimized convolutional layers, the proposed method enhances both feature representation and classification reliability.

In summary, this study makes the following contributions. First, we propose an automated DDH classification framework based on YOLOv11, optimized for real-time ultrasound diagnosis. Second, we introduce a preprocessing pipeline that combines UMAP-based visualization with data augmentation to alleviate class imbalance and enhance model robustness. Third, we integrate advanced spatial attention mechanisms (C2PSA) into the YOLOv11 architecture to strengthen anatomical feature recognition. Finally, we validate the proposed model against lightweight benchmark networks, demonstrating superior accuracy and inference speed suitable for clinical application.

The remainder of this paper is organized as follows. Section 2 reviews related work on DDH diagnosis and deep learning in medical imaging. Section 3 describes the proposed methodology, including dataset acquisition, preprocessing, and model architecture. Section 4 presents experimental results and performance analyses. Section 5 discusses the findings and their clinical implications, and Section 6 concludes the study with future research directions.

## 2. Related Work

### 2.1 Ultrasound-Based DDH Diagnosis and Graf Classification

Due to the inherent challenges of accurately delineating the proximal femur and acetabular margin in neonatal hip X-ray imaging 12, ultrasound has become the preferred modality for diagnosing developmental dysplasia of the hip (DDH) 13. Although its role in large-scale screening programs remains debated 14, ultrasound continues to be widely adopted across Europe 15. Several diagnostic approaches have been developed, including the Graf, Harcke, Terjesen, and Suzuki methods, with the Graf technique being the most widely accepted for screening, diagnosis, and treatment monitoring of DDH 16.

The Graf method relies on predefined anatomical landmarks within the hip joint, identifying five critical points: the iliac outer edge, the lower limb of the ilium, the transition point where the bony acetabular roof curves toward the ilium, the center of the labrum, and the femoral head 17. By constructing three intersecting lines—the baseline, the bony roof line, and the soft tissue covering line—two key angles can be measured: the α angle (bony roof angle) and the β angle (cartilage roof angle) 18, 19. These parameters enable the classification of neonatal hips into distinct categories:

- Type I (Normal Hip): α > 60°
- Type IIa/IIb (Immature Hip): 50° ≤ α ≤ 59°
- Type IIc/D (Dysplastic Hip): α < 50°
- Type III/IV (Dislocated Hip): α < 43° 20-22.

Ultrasound imaging offers significant advantages, including ease of operation, reproducibility, and the absence of ionizing radiation 23. However, the accuracy of the Graf method depends heavily on strict adherence to standardized imaging protocols 24. Failure to capture the standard plane can result in measurement errors and subjective interpretation, thereby reducing the reliability of α and β angle assessments 25, 26. These limitations underscore the importance of developing automated image analysis techniques to improve diagnostic precision and consistency.

### 2.2 Deep Learning for Medical Image Analysis

Deep learning has revolutionized medical image analysis, demonstrating remarkable performance in segmentation, classification, and anomaly detection tasks across diverse clinical domains 27-29. While the concept of artificial neural networks (ANNs) originated in 1943 30, 31, the advent of deep learning in 2006 enabled the development of multi-layer network architectures with enhanced representational capacity 32. Among these, Convolutional Neural Networks (CNNs) have driven major breakthroughs in applications such as disease diagnosis, semantic segmentation, and object detection 33-35.

CNN-based approaches have become the dominant paradigm in medical imaging 36, 37, leveraging hierarchical feature extraction to achieve high precision in identifying and localizing pathological structures 38. However, these methods often require large-scale, expert-labeled datasets, making data annotation labor-intensive and time-consuming 39-41. This challenge highlights the need for scalable, automated, and real-time classification systems that can reduce reliance on manual labeling while maintaining diagnostic accuracy 42, 43.

### 2.3 Deep Learning for DDH Classification

Convolutional Neural Networks (CNNs) have shown encouraging results in ultrasound-based DDH classification, particularly for segmenting femoral head and acetabular structures 44. Early studies primarily employed conventional machine learning techniques; however, the introduction of Fully Convolutional Networks (FCNs), U-Net, and transformer-based architectures has substantially improved segmentation accuracy in recent years 45, 46. Despite these advances, most existing methods continue to emphasize segmentation rather than direct classification of DDH severity.

For reliable classification, access to sufficiently large and high-quality labeled datasets is essential 47. Yet, training on imbalanced datasets frequently results in biased predictions, as underrepresented classes are inadequately learned 48. Moreover, medical ultrasound images often suffer from noise, speckle artifacts, and incomplete anatomical visualization, all of which degrade CNN performance 49, 50. Preprocessing techniques such as noise reduction and data augmentation can mitigate these limitations, but they inevitably increase computational complexity 48-50. These challenges underscore the necessity for more robust, efficient, and clinically applicable classification frameworks tailored to DDH diagnosis.

### 2.4 YOLO-based Medical Image Classification

The You Only Look Once (YOLO) family of models has been widely adopted for real-time object detection and classification across diverse domains, including medical imaging 51. Recent iterations such as YOLOv4, YOLOv5, and YOLOv8 have introduced optimized backbone networks, attention mechanisms, and enhanced feature fusion modules, leading to notable improvements in both accuracy and computational efficiency 52, 53. Unlike conventional CNN-based classification approaches, YOLO simultaneously performs object localization and classification in a single forward pass, making it highly suitable for time-sensitive diagnostic applications.

The latest version, YOLOv11, incorporates Cross-Stage Partial (CSP) connections, C2PSA spatial attention mechanisms, and efficient convolutional blocks, enabling superior feature extraction while maintaining lightweight computational demands 54. This architecture has already been applied successfully in tasks such as chest X-ray interpretation, ultrasound imaging, and medical anomaly detection, where it has consistently outperformed lightweight models such as MobileNet and ShuffleNet in terms of classification accuracy and inference speed 55-62.

Traditional ultrasound-based DDH diagnosis remains dependent on manual interpretation, which is inherently variable across operators. By contrast, YOLO-based automated classification provides a fast, accurate, and scalable alternative, ideally suited for real-time clinical applications. Building on these advances, the present study leverages YOLOv11 for DDH classification, aiming to address persistent challenges such as dataset imbalance, image noise, and diagnostic inconsistency.

## 3. Methodology

### 3.1 Data Acquisition

Ultrasound images used in this study were acquired using a diagnostic ultrasound system. The dataset comprised 6,075 images stored in DICOM format for static frames and AVI format for video sequences. Images were categorized into ten anatomical classes: hip, ankle, soft tissue, wrist, shoulder, finger, knee, elbow, foot, and other.

**Figure 1:**
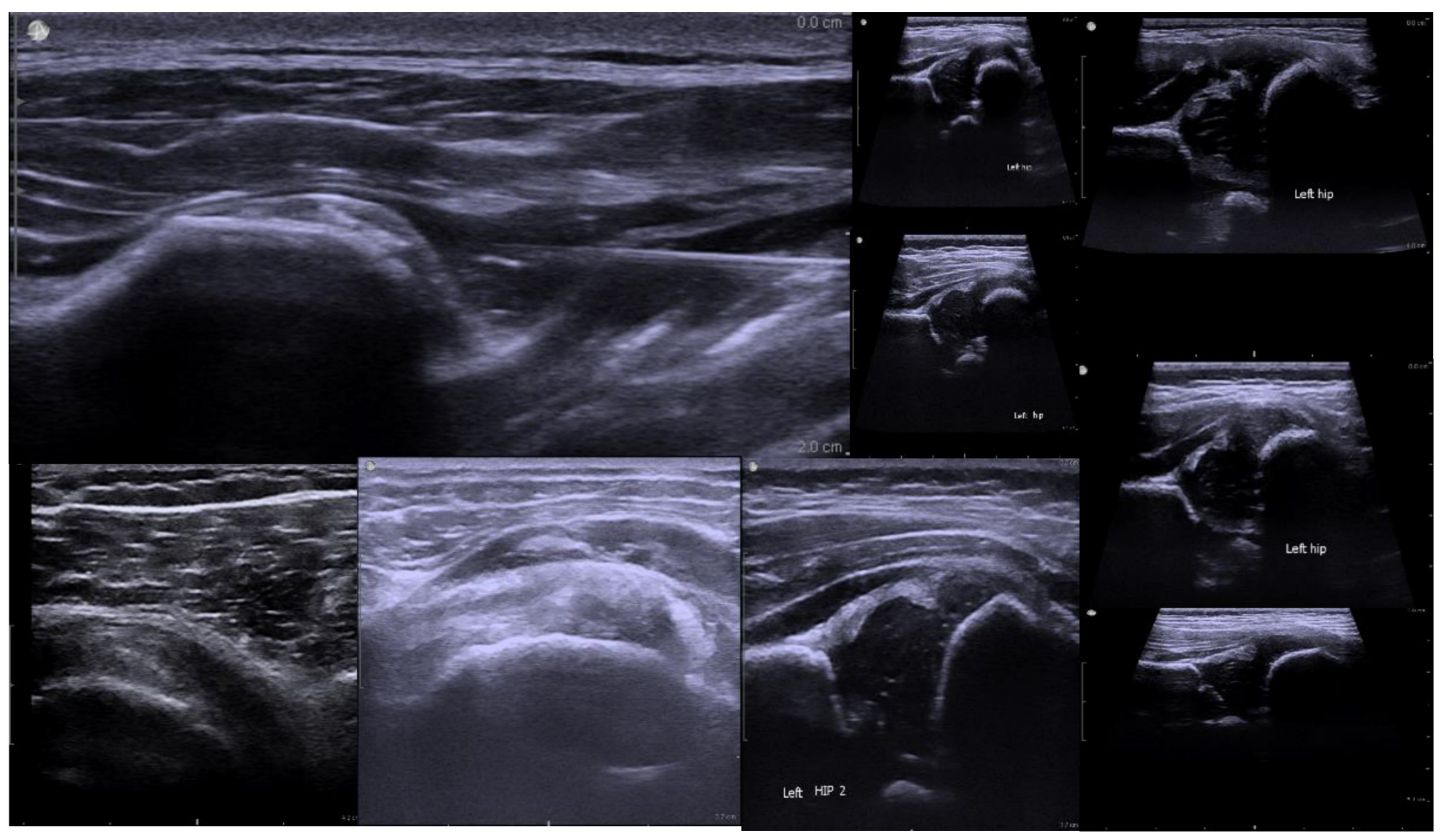
Ultrasound Image of the Hip Joint in Developmental Dysplasia of the Hip (DDH).

For the assessment of Developmental Dysplasia of the Hip (DDH), imaging primarily targeted key anatomical structures including the femoral head, acetabulum, and ilium. The Graf classification method, which evaluates hip joint stability through the measurement of the α and β angles, was adopted as the clinical reference standard. Figure 1 illustrates a representative hip joint ultrasound image, highlighting the femoral head's position within the acetabulum and labeling the femoral head, acetabulum, and ilium—structures essential for determining joint alignment and identifying abnormalities.

The dataset was collected from multiple clinical sources to capture diversity in patient demographics, imaging protocols, and anatomical variations. However, a marked class imbalance was observed: the hip category contained a disproportionately high number of images (5,159), compared with other categories such as knee (13) and ankle (5). This imbalance necessitated the use of data augmentation strategies, described in Section 3.2.

### 3.2 Dataset Analysis and Preprocessing

#### 3.2.1 Dataset Imbalance and Visualization

The dataset exhibited pronounced class imbalance, which can bias model training and compromise generalization performance. To evaluate the distribution of samples, we employed Uniform Manifold Approximation and Projection (UMAP), a state-of-the-art dimensionality reduction technique well-suited for high-dimensional medical imaging data 51. UMAP preserves both local and global data structures, thereby providing a reliable representation of class distribution.

**Figure 2:**
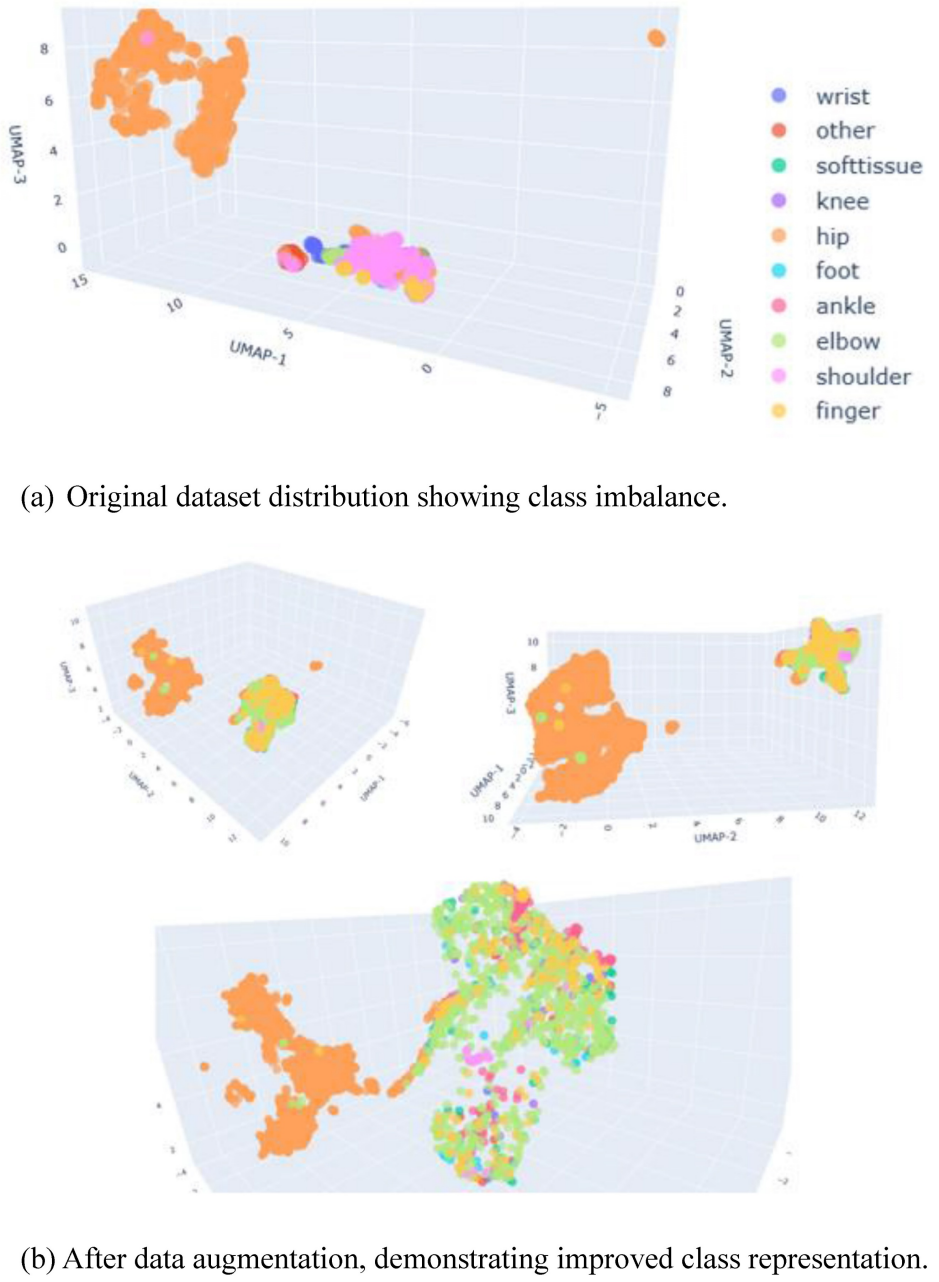
UMAP Visualization of Dataset Distribution.

As illustrated in Figure 2(a), the original dataset was dominated by the hip class, which clustered separately from the other anatomical categories. Following the application of data augmentation, the distribution became more balanced, as shown in Figure 2(b). The numerical breakdown of each class before and after augmentation is presented in Table 1.

#### 3.2.2 Data Augmentation Strategy

To mitigate the effects of imbalance and enhance model robustness, multiple augmentation techniques were applied:

- Geometric Transformations: Random rotations (±15°), translations, and scaling to simulate variability in patient positioning.
- Intensity Adjustments: Gamma correction to normalize differences in brightness and contrast across scans.
- Elastic Deformations: Applied to approximate natural soft tissue variability.
- Noise Injection and Bias Field Distortion: Introduced random artifacts to replicate real-world imaging conditions.

These augmentation strategies improved class balance and promoted better generalization, as demonstrated by the more uniform UMAP distribution shown in Figure 2(b).

### 3.3 Proposed YOLOv11-Based Classification Model

The proposed YOLOv11-based framework was designed to automate DDH classification from ultrasound images while maintaining both high inference speed and diagnostic accuracy.

#### 3.3.1 Model Architecture

**Figure 3:**
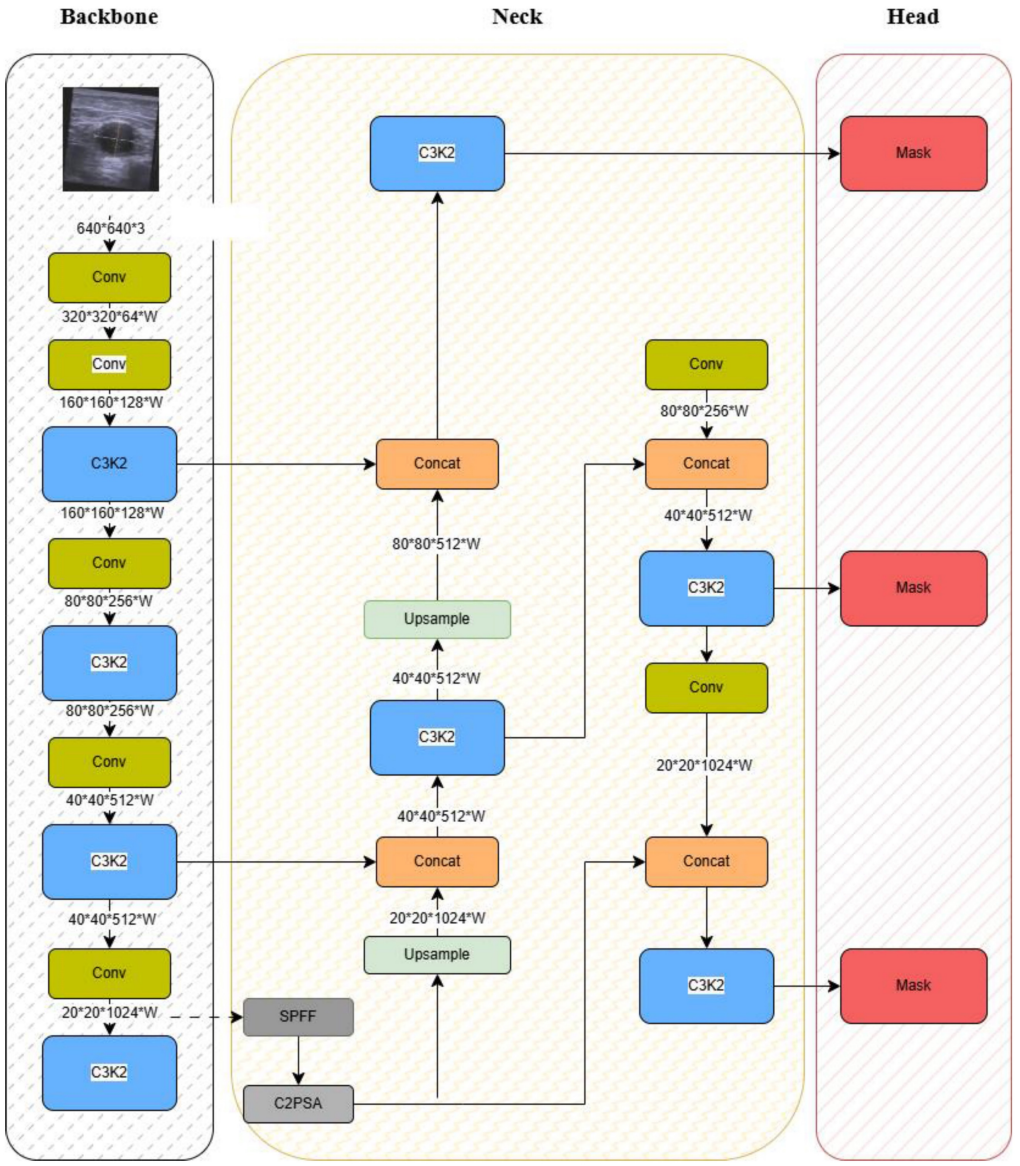
YOLOv11 Model Architecture.

Figure 3 presents an overview of the YOLOv11 architecture adapted for this study. The framework introduces several key innovations:

(1) Backbone (Feature Extraction)

- C3k2 Blocks: An efficient implementation of Cross-Stage Partial (CSP) bottlenecks, improving gradient flow and feature reuse 52.
- Spatial Pyramid Pooling - Fast (SPPF): Reduces computational cost while retaining multi-scale feature representation.
- C2PSA Attention Mechanism: Enhances spatial feature learning, enabling the model to focus on clinically relevant anatomical regions.

(2) Neck (Feature Aggregation)

- Combines Feature Pyramid Network (FPN) and Path Aggregation Network (PAN) structures.
- C3k2 blocks replace traditional C2f blocks, yielding higher efficiency without compromising accuracy

(3) Head (Prediction)

- Outputs bounding boxes, class probabilities, and confidence scores in a single forward pass, thereby supporting real-time classification.

As shown in Figure 3, these components work synergistically to extract discriminative anatomical features, aggregate multi-scale information, and deliver accurate classification under real-time constraints. The integration of CSP and C2PSA modules is particularly critical for modeling complex hip structures in noisy ultrasound environments.

Together, these architectural improvements enable the YOLOv11 framework to achieve both high diagnostic accuracy and computational efficiency, rendering it suitable for deployment in point-of-care ultrasound systems.

### 3.4 Training Procedure

The training procedure for the YOLOv11-based classification model consisted of data preprocessing, model training, hyperparameter optimization, and performance evaluation. The pipeline was designed to maximize generalization, minimize overfitting, and ensure robust classification across imbalanced classes.

#### 3.4.1 Model Training and Optimization

The model was trained to simultaneously optimize feature extraction, localization, and classification. Training was conducted using the AdamW optimizer, which balances convergence speed and generalization by incorporating weight decay regularization. A cosine annealing learning rate schedule was applied, beginning with an initial learning rate of 0.001 and gradually decaying to stabilize learning and reduce overfitting.

To address class imbalance, Focal Loss was employed, reducing the influence of easily classified samples while emphasizing harder-to-classify cases. For localization refinement, IoU Loss was used to improve bounding box predictions and ensure accurate delineation of hip joint structures. The final training configuration was as follows:

- Initial Learning Rate: 0.001 (cosine decay)
- Batch Size: 16
- Number of Epochs: 100
- Optimizer: AdamW with weight decay = 0.01
- Loss Functions: (1) Focal Loss for classification, (2) IoU Loss for localization

To improve generalization, the dataset was augmented with random rotations, brightness adjustments, and noise injection, as described in Section 3.2.2. Importantly, patient-level data splitting was applied to prevent information leakage: all images from the same patient were assigned exclusively to one partition. An 80/20 split at the patient level was used for development (training/validation), followed by 5-fold cross-validation, also stratified by patient, to ensure robustness. This design eliminated identical-patient overlap across folds, providing an unbiased estimate of model generalization.

#### 3.4.2 Evaluation Metrics

Model performance was evaluated using four standard metrics:

- Accuracy (ACC): Proportion of correctly classified cases among all predictions.

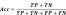

- Precision (P): Proportion of correctly identified positive cases among all predicted positives, reflecting the ability to avoid false positives.

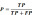

- Recall (R): Proportion of correctly identified positive cases among all actual positives, reflecting sensitivity and the ability to reduce false negatives.

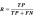

- F1-Score: Harmonic mean of precision and recall, providing a balanced measure of model performance.

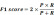



The proposed model was benchmarked against lightweight classification architectures, MobileNetV3 and ShuffleNetV2, with comparisons focusing on accuracy, inference time, and computational efficiency. In addition, 5-fold cross-validation was performed to validate the consistency and robustness of results across different dataset partitions.

## 4. Results and Discussion

### 4.1 Performance Comparison

The YOLOv11-based DDH classification model was first evaluated on the independent test set and compared with two lightweight baseline architectures, MobileNetV3 and ShuffleNetV2. Performance was assessed using four metrics: Accuracy, Precision, Recall, and F1-Score. The results are summarized in Table 2.

As shown in Table 2, YOLOv11 achieved the highest performance across all metrics, with an overall accuracy of 95.05%. This represents a substantial improvement of nearly 20% in accuracy compared with MobileNetV3 (75.6%) and more than 23% compared with ShuffleNetV2 (71.9%). Precision and recall values for YOLOv11 were also consistently higher, resulting in the highest F1-Score (95.05%).

These results highlight the effectiveness of incorporating CSP and C2PSA modules into the YOLOv11 architecture, which improved the model's capacity to capture clinically relevant anatomical structures in ultrasound images. The superior performance across multiple evaluation metrics demonstrates that the proposed framework not only outperforms lightweight alternatives but also provides reliable classification suitable for real-time clinical deployment.

### 4.2 Confusion Matrix Analysis

**Figure 4:**
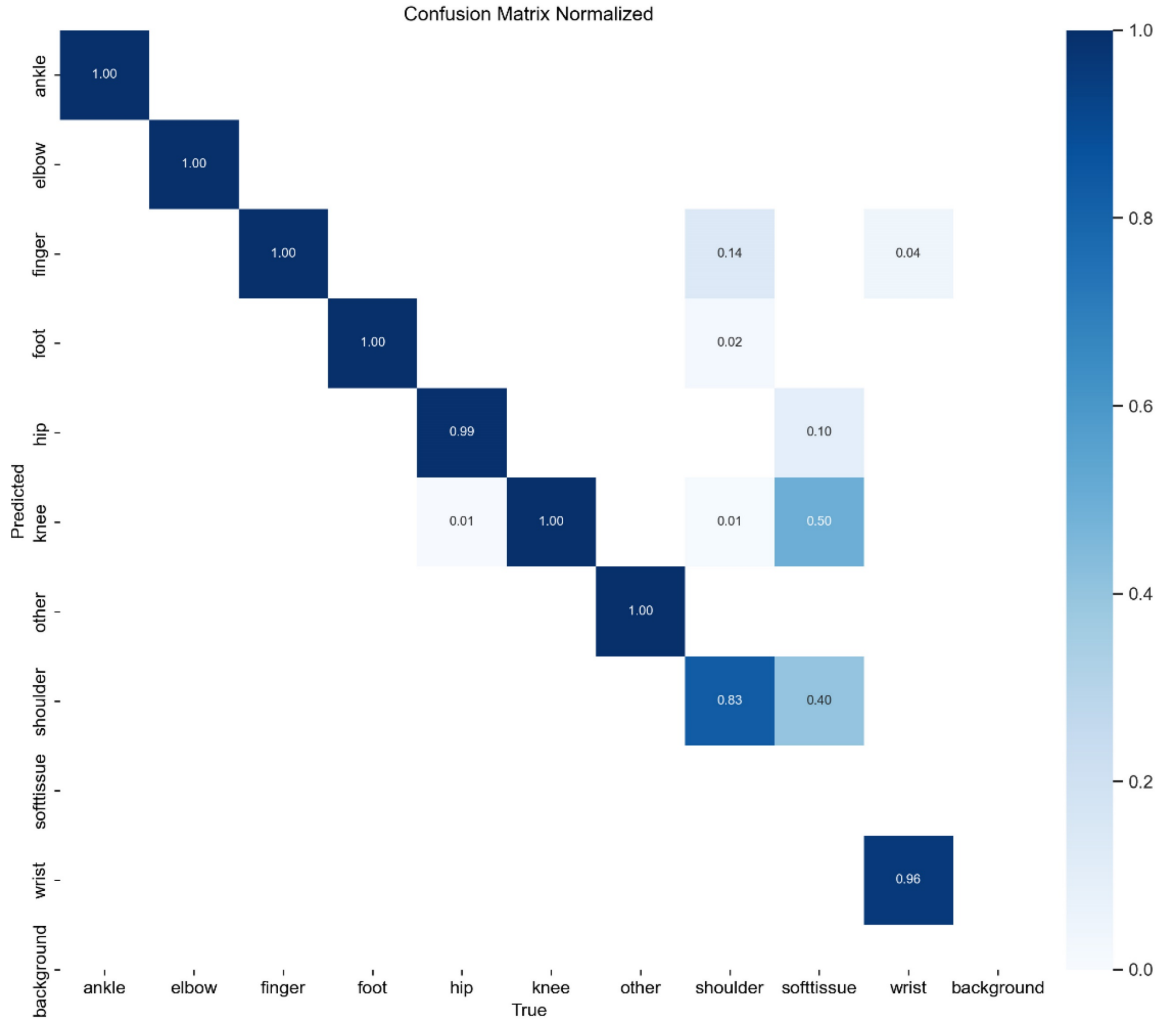
Confusion Matrix of YOLOv11 Model.

To further assess classification performance, a confusion matrix was generated for YOLOv11, as presented in Figure 4. The matrix illustrates the distribution of correct and incorrect predictions across different DDH categories, providing detailed insights into class-specific strengths and weaknesses.

**Figure 5:**
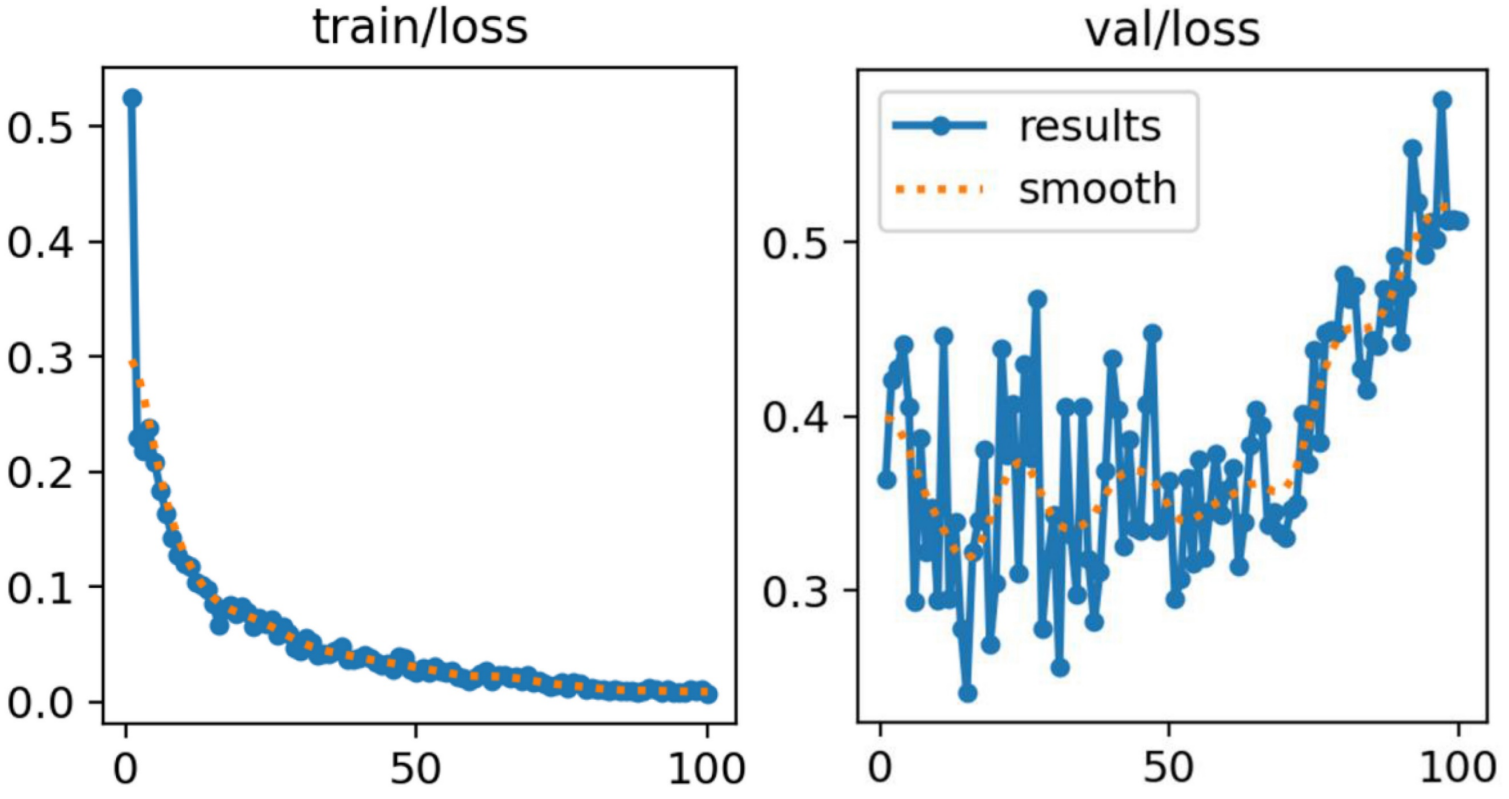
Training and Validation Loss Curves for YOLOv11.

As shown in Figure 5, both training and validation losses decreased steadily throughout the training process, with no evidence of divergence between the two curves. This pattern indicates stable learning dynamics and suggests that overfitting was effectively mitigated. The application of data augmentation and regularization strategies contributed to this stability by improving generalization and reducing susceptibility to noise or class imbalance.

The consistent convergence observed in both curves demonstrates that the model successfully captured discriminative features of hip anatomy without sacrificing generalization capacity. These results further validate the suitability of the proposed training strategy, confirming its robustness in handling heterogeneous ultrasound data.

Overall, the model achieved high classification accuracy across the majority of categories. However, some degree of misclassification was observed. Specifically, overlap occurred between Type IIa and Type IIb hips, reflecting their inherent anatomical similarity and the subtle differences in α angle measurements that challenge even experienced clinicians. In addition, certain Type III cases were misclassified as Type IIc, likely due to similarities in femoral head positioning.

These findings indicate that while the model performs robustly overall, borderline categories remain the most challenging to differentiate. This limitation parallels the clinical reality, where even expert sonographers occasionally encounter difficulties distinguishing between adjacent Graf types. Such results suggest that additional strategies, such as refined preprocessing, multi-view ultrasound integration, or hybrid AI-physician decision-making could further improve classification accuracy in these borderline cases.

### 4.3 Training and Validation Loss Analysis

The training and validation loss curves for the YOLOv11 model are presented in Figure 5. These curves depict the optimization trajectory across 100 epochs, illustrating both convergence behavior and generalization performance.

### 4.4 Inference Speed and Computational Efficiency

Inference efficiency is a critical factor for real-time DDH screening. Table 3 compares the number of parameters, FLOPs, and inference time per image for YOLOv11, MobileNetV3, and ShuffleNetV2.

Although YOLOv11 contains a larger number of parameters (12.9M) and higher computational complexity (49.4B FLOPs) compared with MobileNetV3 and ShuffleNetV2, its inference speed remained competitive at 11.5 ms per image. This demonstrates that the architectural optimizations—including CSP modules, spatial attention mechanisms, and efficient convolutional blocks—effectively balanced accuracy with efficiency.

The results confirm that YOLOv11 is capable of achieving real-time performance without compromising diagnostic precision. This balance between computational cost and inference speed makes the framework well-suited for integration into point-of-care ultrasound systems, where rapid diagnostic feedback is essential.

### 4.5 Comparison with Previous Studies

To contextualize the performance of the proposed framework, we compared it against prior deep learning approaches for DDH classification. Table 4 summarizes the results.

As shown in Table 4, YOLOv11 achieved a classification accuracy of 95.05%, outperforming earlier CNN- and ResNet-based models by a margin of 6-8%. This improvement can be attributed to three factors: (i) the use of a larger dataset collected over multiple years, (ii) architectural enhancements such as CSP modules and C2PSA spatial attention, and (iii) robust data augmentation strategies that alleviated class imbalance.

Compared with earlier approaches, which primarily emphasized segmentation or employed conventional CNN backbones, the proposed model provides a more scalable and clinically practical solution. Its superior accuracy, combined with real-time inference speed, underscores its potential for deployment in routine DDH screening workflows.

### 4.6 Ablation Study

To evaluate the individual contributions of the Cross-Stage Partial (CSP) modules and the C2PSA spatial attention mechanism, an ablation study was conducted under four experimental settings: (1) YOLOv11 without CSP, (2) YOLOv11 without C2PSA, (3) YOLOv11 without both modules, and (4) the full model (CSP + C2PSA). The results are summarized in Table 5.

As shown in Table 5, removal of either CSP or C2PSA resulted in a noticeable decline in performance compared with the full model. Excluding CSP primarily reduced recall, indicating its importance for enhancing sensitivity in detecting dysplastic hips. In contrast, the absence of C2PSA led to lower precision, suggesting that spatial attention was critical for guiding the network toward anatomically relevant regions and minimizing false positives. The combined use of CSP and C2PSA produced the best overall performance, underscoring their complementary roles in improving both feature extraction and anatomical interpretability.

These findings confirm that the architectural modifications introduced in YOLOv11 are not only computationally efficient but also essential for achieving clinically meaningful performance in DDH classification.

### 4.7 Explainability Analysis

**Figure 6:**
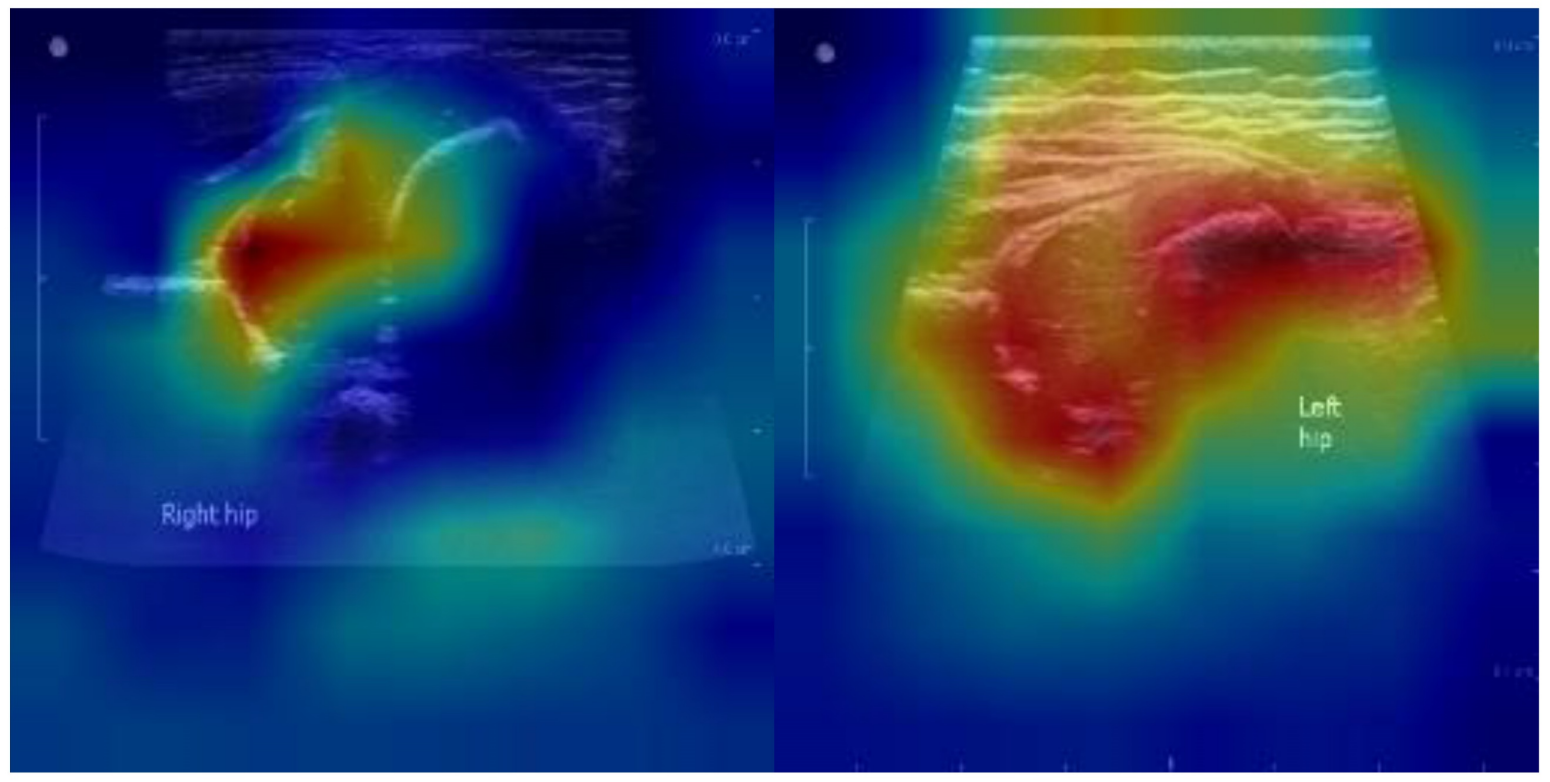
**Grad-CAM visualizations of YOLOv11-based DDH classification.** (a) Normal hip: the model predominantly focuses on the acetabular roof and femoral head. (b) Dysplastic hip: stronger activation is observed around the shallow acetabular roof and displaced femoral head. These attention patterns align with Graf classification landmarks, supporting clinical interpretability and acceptance.

To improve interpretability and foster clinical acceptance, we generated Gradient-weighted Class Activation Mapping (Grad-CAM) and attention heatmaps for representative cases. As illustrated in Figure 6, the model consistently focused on the acetabular roof and femoral head—key anatomical landmarks central to the Graf classification system.

In normal hips, Grad-CAM highlighted concentrated attention on the acetabular roof and femoral head, confirming that the model relies on clinically relevant structures for accurate classification. In dysplastic hips, stronger activations were observed around the shallow acetabular roof and the displaced femoral head, patterns that align closely with radiologists' visual assessments.

These findings suggest that the model's decision-making process is not only data-driven but also anatomically meaningful. By attending to regions routinely evaluated by clinicians, the proposed framework enhances transparency and builds trust, thereby facilitating its potential integration into real-time, point-of-care diagnostic workflows.

### 4.8 Clinical Workflow Integration

To facilitate clinical adoption, we envision several pathways through which the proposed model could be integrated into routine workflows across different care settings.

Neonatal screening clinics. During routine hip ultrasound examinations, the system can be embedded directly within the ultrasound console or a connected edge device. It provides (i) real-time quality feedback, such as alerts for non-standard planes or probe instability, (ii) live classification overlays to support immediate triage (normal vs. dysplastic/immature), and (iii) structured outputs including key frames and confidence scores for documentation. This integration reduces the need for repeat scans, shortens examination time, and enhances diagnostic efficiency in high-volume screening settings.

Primary healthcare and community hospitals. In resource-limited or non-specialist environments, the framework can function as a decision-support tool. Cases classified with low confidence are flagged for secondary review by pediatric orthopedists, while high-confidence normal cases may be safely discharged with follow-up instructions. This hub-and-spoke model optimizes referral pathways, reduces unnecessary specialist consultations, and ensures that expert attention is focused on the most complex cases.

Training and quality assurance. Explainability features, such as Grad-CAM heatmaps, provide immediate feedback by highlighting clinically relevant structures (e.g., acetabular roof, femoral head). These visualizations can be used as teaching aids for junior sonographers and as part of standardized training programs. From a quality assurance perspective, periodic audits of flagged cases and drift monitoring can be implemented to ensure sustained accuracy after deployment.

Human-in-the-loop safeguards. The model is designed to complement, not replace, physician expertise. Safety features include threshold-based alerts, confidence-calibrated reporting, and mandatory human review for ambiguous or low-confidence cases. These safeguards ensure that final diagnostic responsibility remains with clinicians while leveraging AI to improve efficiency and consistency.

## 5. Discussion

The proposed YOLOv11-based framework demonstrates both technical innovation and clinical applicability for automated DDH classification.

From a technical perspective, several architectural enhancements directly contributed to the model's superior performance. The incorporation of Cross-Stage Partial (CSP) blocks improved gradient flow and feature reuse, thereby enhancing recall and sensitivity for detecting dysplastic hips. The addition of the C2PSA spatial attention mechanism enabled the model to focus on anatomically meaningful regions, reducing false positives and improving precision. Results from the ablation study confirmed the complementary roles of these modules, with the full model achieving the highest overall accuracy (95.05%) and real-time inference speed (11.5 ms per image). When compared with lightweight baselines such as MobileNetV3 and ShuffleNetV2, the proposed framework consistently demonstrated superior performance across multiple metrics, underscoring the importance of architectural optimization. Moreover, the integration of Focal Loss and IoU Loss effectively addressed challenges related to class imbalance and localization, ensuring stable training and robust generalization.

From a clinical perspective, the framework represents an important step toward standardizing DDH screening, which remains subject to significant inter-observer variability under the Graf classification system. By enabling real-time automated classification, the model can support clinicians in neonatal screening clinics and community healthcare settings, where operator expertise is often limited. Potential applications include immediate feedback during scanning, triage support through abnormal case flagging, and automated report generation to reduce documentation burden. Importantly, explainability analyses such as Grad-CAM demonstrated that the model consistently focused on the acetabular roof and femoral head, key anatomical landmarks used in clinical practice. This alignment with established diagnostic criteria enhances transparency, fosters clinician trust, and strengthens the case for clinical adoption.

Despite these strengths, several limitations must be acknowledged. First, although the dataset comprised more than 6,000 ultrasound images, all data were obtained from a single institution. This may restrict generalizability due to variations in imaging equipment, acquisition protocols, and patient demographics. To address this limitation, future work will emphasize external validation across multiple centers and populations. Second, although patient-level data splitting was applied to eliminate information leakage, prospective clinical validation remains necessary to fully assess performance in real-world workflows. Finally, while ultrasound is the gold standard for infant DDH screening, incorporating multimodal imaging modalities such as X-ray and MRI could broaden diagnostic capability and improve precision.

In summary, the proposed YOLOv11 framework integrates ablation-validated architectural innovations, real-time feasibility, and clinically meaningful explainability. Its potential applications extend beyond technical accuracy to address practical challenges in neonatal screening and primary care environments. Future research should prioritize multi-center validation, prospective deployment in point-of-care ultrasound systems, and multimodal integration to ensure robust clinical translation and maximize the framework's impact in standardized DDH diagnosis.

## 6. Conclusion

In this study, we developed an attention-enhanced YOLOv11 framework for the automated classification of developmental dysplasia of the hip (DDH) from ultrasound images. By integrating Cross-Stage Partial (CSP) modules and C2PSA spatial attention, the model achieved superior performance, with an accuracy of 95.05% and an inference speed of 11.5 ms per image. Ablation experiments confirmed the complementary roles of CSP and C2PSA, demonstrating their collective impact on improving both sensitivity and precision.

Beyond technical performance, the framework provides tangible clinical benefits. Real-time classification and interpretable visualizations, supported by Grad-CAM heatmaps, align with established diagnostic landmarks such as the acetabular roof and femoral head. These features enhance transparency, reduce inter-observer variability, and facilitate integration into neonatal screening clinics and community healthcare settings, particularly where operator expertise may be limited.

To ensure unbiased evaluation, patient-level data splitting was employed to prevent information leakage, providing a reliable estimate of clinical performance. Nevertheless, the reliance on a single-institution dataset remains a limitation. Future work will focus on multi-center external validation, prospective deployment within point-of-care ultrasound systems, and multimodal integration (e.g., X-ray and MRI) to further expand diagnostic capability and generalizability.

In conclusion, this work demonstrates both technical innovation and clinical practicality. By combining ablation-validated architectural improvements, explainability, and workflow-oriented design, the proposed YOLOv11 framework establishes a foundation for clinically deployable, AI-assisted DDH screening. Future research directed toward multi-center validation and multimodal expansion will be essential for translating this framework into standardized clinical practice and maximizing its impact in pediatric orthopedic care.

## Figures and Tables

**Table 1:** Dataset Distribution Before and After Augmentation.

Class	Original Training Set	Original Test Set	Augmented Training Set	Augmented Test Set
Hip	5,159	573	5,159	573
Shoulder	178	19	890	95
Knee	13	4	56	20
Ankle	5	1	25	5
Wrist	31	5	155	25
Finger	14	3	70	15
Elbow	12	4	60	20
Foot	4	1	20	5
Soft Tissue	5	4	20	20

**Table 2:** Performance Comparison of YOLOv11, MobileNetV3, and ShuffleNetV2.

Model	Accuracy (%)	Precision (%)	Recall (%)	F1-Score (%)
YOLOv11	**95.05**	**94.88**	**95.22**	**95.05**
MobileNetV3	75.6	76.1	74.5	75.3
ShuffleNetV2	71.9	72.4	70.8	71.6

**Table 3:** Inference speed and computational efficiency comparison.

Model	Parameters (M)	FLOPs (B)	Inference Time (ms/image)
YOLOv11	12.9	49.4	11.5
MobileNetV3	5.4	2.19	12.0
ShuffleNetV2	2.3	1.46	11.4

**Table 4:** Comparison with Existing DDH Classification Models.

Study	Model Used	Accuracy (%)	Dataset Size
Sezer et al. (2020) 13	CNN + Data Augmentation	87.3	2,500 images
Chlapoutakis et al. (2022) 24	ResNet-50	89.1	3,200 images
This Study	YOLOv11	95.05	6,075 images

**Table 5:** Ablation Study of CSP and C2PSA Modules.

Model Variant	Accuracy (%)	Precision (%)	Recall (%)	F1-Score (%)
YOLOv11 without CSP	91.8	91.2	91.5	91.3
YOLOv11 without C2PSA	92.6	92.1	92.3	92.2
YOLOv11 without CSP + C2PSA	90.9	90.3	90.7	90.5
Full Model (CSP + C2PSA)	**95.05**	**94.88**	**95.22**	**95.05**
